# Novel Aflatoxin-Degrading Enzyme from *Bacillus shackletonii* L7

**DOI:** 10.3390/toxins9010036

**Published:** 2017-01-14

**Authors:** Liang Xu, Mohamed Farah Eisa Ahmed, Lancine Sangare, Yueju Zhao, Jonathan Nimal Selvaraj, Fuguo Xing, Yan Wang, Hongping Yang, Yang Liu

**Affiliations:** 1 Institute of Food Science and Technology, Chinese Academy of Agricultural Sciences, 1 Nongda South Road, Xibeiwang Town, Haidian District, Beijing 100193, China; xuliang19824@163.com (L.X.); elfarahy89@hotmail.com (M.F.E.A.); lancin.sangar@gmail.com (L.S.); zhaoyueju@caas.cn (Y.Z.); sjonnim@gmail.com (J.N.S.); xingfuguo@caas.cn (F.X.); awangyan@126.com (Y.W.); 2 Key Laboratory of Agro-products Processing, Ministry of Agriculture, 1 Nongda South Road, Xibeiwang Town, Haidian District, Beijing 100193, China; 3 Shenyang Institute of Engineering, No.18 Puchang Road, Shenbei New District, Shenyang 110136, China; yanghp@sie.edu.cn

**Keywords:** aflatoxin B_1_, aflatoxin-degrading enzyme, biodegradation, *Bacillus shackletonii*, purification

## Abstract

Food and feed contamination by aflatoxin (AF)B_1_ has adverse economic and health consequences. AFB_1_ degradation by microorganisms or microbial enzymes provides a promising preventive measure. To this end, the present study tested 43 bacterial isolates collected from maize, rice, and soil samples for AFB_1_-reducing activity. The higher activity was detected in isolate L7, which was identified as *Bacillus shackletonii*. L7 reduced AFB_1_, AFB_2_, and AFM_1_ levels by 92.1%, 84.1%, and 90.4%, respectively, after 72 h at 37 °C. The L7 culture supernatant degraded more AFB_1_ than viable cells and cell extracts; and the degradation activity was reduced from 77.9% to 15.3% in the presence of proteinase K and sodium dodecyl sulphate. A thermostable enzyme purified from the boiled supernatant was designated as Bacillus aflatoxin-degrading enzyme (BADE). An overall 9.55-fold purification of BADE with a recovery of 39.92% and an activity of 3.85 × 10^3^ U·mg^−1^ was obtained using chromatography on DEAE-Sepharose. BADE had an estimated molecular mass of 22 kDa and exhibited the highest activity at 70 °C and pH 8.0, which was enhanced by Cu^2+^ and inhibited by Zn^2+^, Mn^2+^, Mg^2+^, and Li^+^. BADE is the major protein involved in AFB_1_ detoxification. This is the first report of a BADE isolated from *B. shackletonii*, which has potential applications in the detoxification of aflatoxins during food and feed processing.

## 1. Introduction

Aflatoxins (AFs) are secondary metabolites produced by *Aspergillus* species, mainly *A. flavus* and *A. parasiticus* [1], that are carcinogenic, teratogenic, and hepatotoxic to both humans and animals, which has grave economic and health consequences [2,3,4]. AFB_1_, the most abundantly produced isotype, is highly toxic [5,6]. Cytochrome P450-associated enzymes in animal liver can metabolize AFB_1_ to AFM_1_, which is detected in the milk of dairy cows that consume AFB_1_-infected feeds [7,8].

Methods for decontamination and reduction of AFs in food sources have been widely investigated. AF inactivation by physical and chemical means is not highly effective or economically feasible [6]; a promising alternative approach is biological detoxification of AF-contaminated food and feed. AFB_1_ biodegradation by fungi and bacteria or their secondary metabolites or enzymes has been widely reported using *Nocardia corynebacterioides* (formerly *Lavobacterium aurantiacum*) [9,10], *Armillariella tabescens* [11,12], *Pleurotus ostreatus* [13], *Bacillus licheniformis* [14], *Mycobacterium fluoranthenivorans* [15,16], *Rhodococcus erythropolis* [16,17,18], *Stenotrophomonas maltophilia* [19], *Myxococcus fulvus* [20], and *Bacillus subtilis* ANSB060 [21]. This approach has the advantage of being highly target-specific, effective, and environmentally safe, as the decontaminated food or feed products can be subsequently used [20].

AF-degrading enzymes have been isolated from a variety of microorganisms. Recently, an aflatoxin oxidase from *A. tabescens* [12] and manganese peroxidase from the white-rot fungus *Phanerochaete sordida* YK-624 [22] were shown to have AFB_1_-degrading ability. It was also reported that a recombinant *Trametes versicolor* laccase enzyme expressed in *Aspergillus niger* degrades AFB_1_ [23]. Nine *Mycobacterium smegmatis* enzymes belonging to two F_420_H_2_-dependent reductase families were found to catalyze the reduction of the α, β-unsaturated ester moiety of AFs by spontaneous hydrolysis [24]. However, most of these enzymes are intracellular and have been isolated from fungi. The process of crushing mycelia to recover enzymes can compromise their activity, preventing their large-scale production. This problem can be circumvented by obtaining AF-degrading enzymes from bacteria.

To this end, a screening method was developed in the present study to isolate AFB_1_-degrading microbes from soils and contaminated kernels using coumarin medium. Several new AFB_1_-degrading bacterial strains were thus identified; among them, *Bacillus shackletonii* strain L7 showed the strongest activity. We evaluated the degradation efficiency of strain L7 against various AFs and purified and characterized a thermostable enzyme named Bacillus aflatoxin-degrading enzyme (BADE) responsible for AFB_1_ degradation activity. We also analyzed the genotoxicity of AFB_1_ degradation products treated with proteins from strain L7.

## 2. Results

### 2.1. Screening for AFB_1_-Degrading Microorganisms

In total, 43 single-colony bacterial isolates were obtained from 247 samples collected from different sources, all of which were able to reduce AFB_1_ to varying degrees in nutrient broth (NB) after incubation for 72 h at 37 °C (Table S1). Eight of the isolates (belonging to the genera *Stenotrophomonas*, *Pseudomonas*, *Arthrobacter*, *Bacillus*, and the family *Flavobacteriaceae*) showed more than 70% AFB_1_ degradation in the medium, with the highest value (71.7%) observed for isolate L7.

### 2.2. Identification of Isolate L7

Isolate L7 was a rod-shaped Gram-positive bacterium that could grow under extreme conditions (55 °C and pH 9.0). It utilized simple sugars like glucose, maltose, and sucrose as a sole carbon source, and hydrolyzed casein but not gelatin, amylum, butyrin, or Tween 80 (Table S2). Based on a 16S rRNA gene sequence analysis, L7 was identified as *Bacillus shackletonii* strain LMG 18435 (99% sequence similarity). This is the first report of a bacterium of this genus exhibiting mycotoxin-degrading ability. The partial 16S rRNA gene sequence of L7 was submitted to GenBank (access. no. KX364157), and the strain was deposited at the China General Microbiological Culture Collection Center (CGMCC8868).

### 2.3. Degradation of AFs by Isolate L7

**Figure 1:**
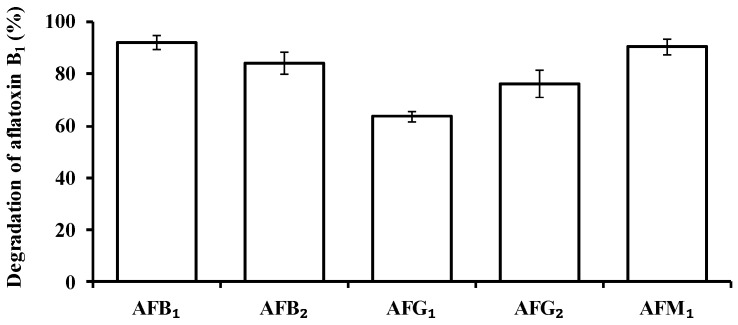
AF degradation by isolate L7. Values represent the means of three replicates and their standard errors.

The degradation activity of isolate L7 towards AFB_1_, AFB_2_, AFG_1_, AFG_2_, and AFM_1_ was 92.1%, 84.1%, 63.6%, 76.1%, and 90.4%, respectively, when cultured in NB at 37 °C for 72 h (Figure 1).

**Figure 2:**
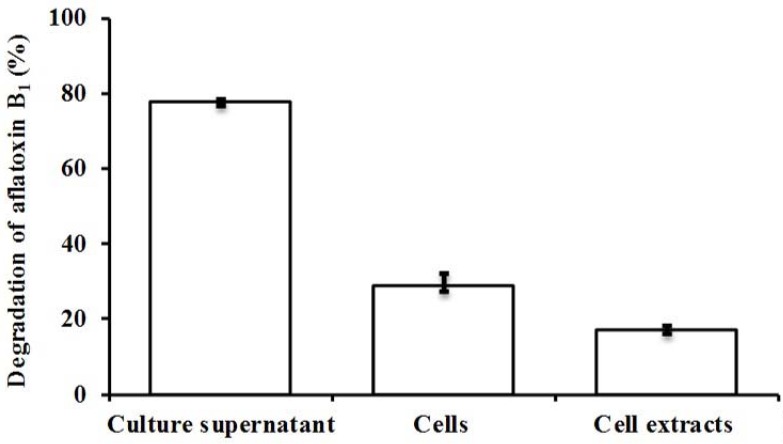
AFB_1_ degradation by L7 culture supernatant, viable cells, and cell extracts after 72 h of incubation. Values represent the means of three replicates and their standard errors.

**Figure 3:**
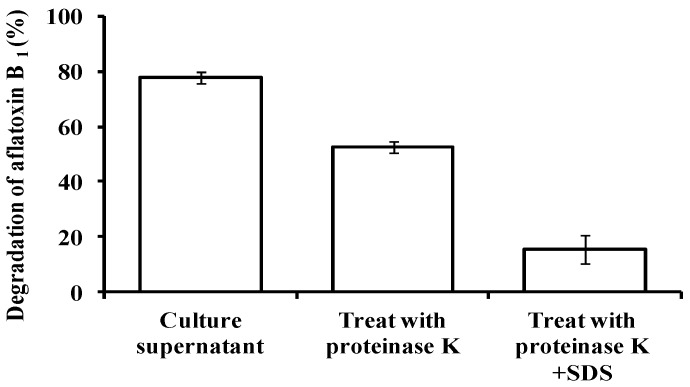
Effect of proteinase K and SDS on AFB_1_ degradation by L7 culture supernatant. Values represent means of three replicates and their standard errors.

The culture supernatant of isolate L7 was more effective than viable cells and cell extracts in degrading higher concentrations of AFB_1_ after 72 h (77.9% vs. 28.6% and 17.2%, respectively; *p* < 0.05) (Figure 2). The AFB_1_-degrading ability of the supernatant declined to 52.6% and 15.3% upon treatment with proteinase K without and with sodium dodecyl sulphate (SDS), respectively (Figure 3). These results suggest that proteins/enzymes secreted by L7 are involved in AFB_1_ degradation.

**Figure 4:**
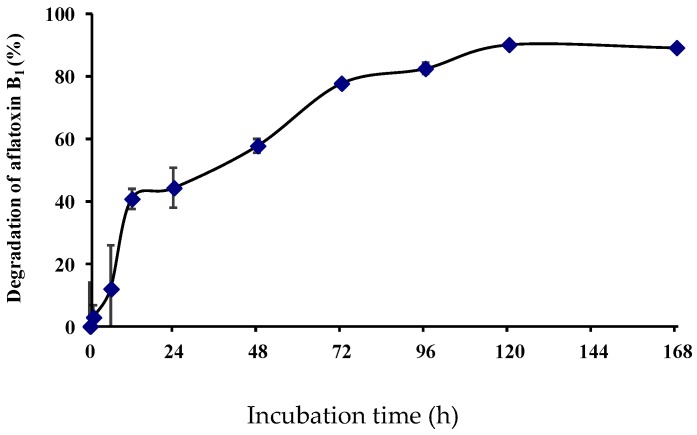
Dynamics of AFB_1_ degradation by isolate L7 culture supernatant at indicated time points. Values represent the means of three replicates and their standard errors.

AFB_1_ degradation by the culture supernatant of isolate L7 proceeded relatively rapidly and continuously, with 40.9, 77.9, and 90.3% reduction observed in the first 12 h and after 72 h and 5 days, respectively (Figure 4).

### 2.4. Evaluation of Genotoxicity

The genotoxicity of the samples was evaluated using the SOS Chromotest, with the results expressed as induction factor ± standard deviation (Table 1). The induction factors of degraded samples incubated in the dark at 37 °C for 72 h with the culture supernatant of isolate L7 were in line with the negative control but not the positive control. At higher concentrations (25, 50, and 100%), the differences between the positive control and degraded samples were significant (*p* < 0.05). These results indicate that AFB_1_ treated with the culture supernatant of isolate L7 had no genotoxicity in the presence of the S9 crude enzyme preparation.

### 2.5. Preliminary Characterization of B. shackletonii BADE

The AFB_1_-degrading ability of the culture supernatant after incubation for 24 h increased from 47.6% to 70.1% upon concentration by 50-fold by ultrafiltration and was positively correlated with protein concentration (Table 2). When the ultrafiltered culture supernatant was heat-treated (incubated in a boiling water bath for 10 min), there was no decrease in degradation activity (76.7%). These results imply that heat-stable proteins or enzymes in the L7 culture supernatant are responsible for AFB_1_ degradation.

### 2.6. BADE Purification

**Figure 5:**
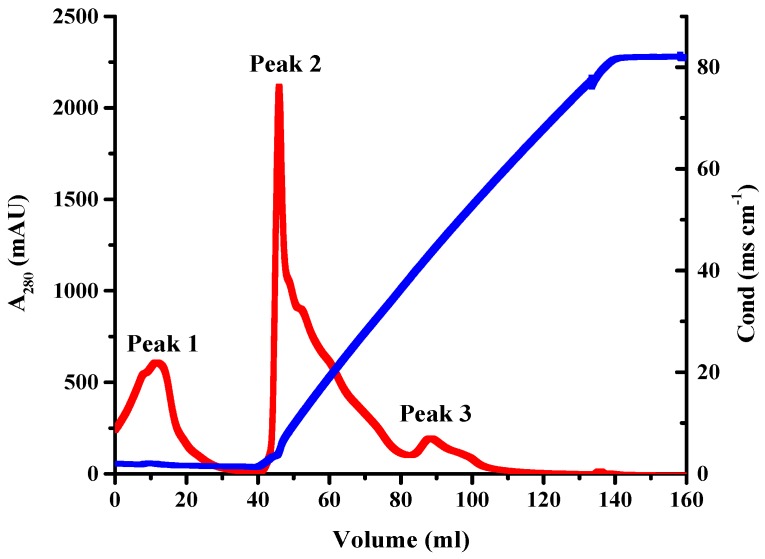
Ion-exchange chromatography using a DEAE-sepharose column. Red and blue lines represent the absorption of the protein at 280 nm and conductance value, respectively. Peak 1 corresponds to the flow-through fraction, and peaks 2 and 3 to the eluted protein.

**Figure 6:**
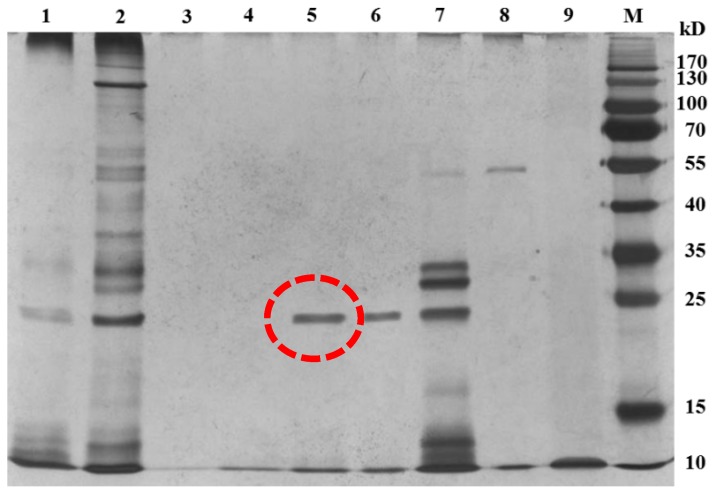
SDS-PAGE gels of proteins obtained during BADE purification. M, protein marker; lane 1, crude protein in culture supernatant of isolate L7 after heat treatment for 10 min; lane 2, crude protein in culture supernatant; lanes 3 and 4, protein in the first flow-through fraction (0–20 mL; peak 1) obtained by ion-exchange chromatography; lanes 5 and 6, protein in the first eluted fraction (40–50 mL, peak 2); lane 7, protein in the peak 2 fraction (50–80 mL); lane 8, protein in the second eluted fraction (80–95 mL, peak 3); lane 9, protein in the peak 3 fraction (95–100 mL).

To isolate the BADE responsible for AFB_1_ degradation, L7 culture supernatant was heat-treated (incubation in a boiling water bath for 10 min), concentrated by ultrafiltration with a cut-off molecular weight of 3 kDa, and subjected to diethylaminoethanol (DEAE)-sepharose ion-exchange chromatography. The maximum absorption of BADE was observed between 240 and 300 nm. The protein concentration of each fraction was monitored at 280 nm during enzyme purification. The separation profile of the enzyme showed one flow-through fraction (peak 1) and two eluted protein peaks (peaks 2 and 3), and different fractions were collected and dialyzed for 24 h against 20 mmol·L^−1^ phosphate buffer (pH 7.4). Compared to the control (AFB_1_ with 20 mmol·L^−1^ phosphate buffer (pH 7.4)), peak 2 (40–50 mL) was associated with the highest AFB_1_-degrading activity (3.85 × 10^3^ U·mg^−1^) (Figure 5), followed by peak 1 (0–20 mL) (2.63 × 10^3^ U·mg^−1^), peak 2 (50–80 mL) (2.39 × 10^3^ U·mg^−1^), peak 3 (80–95 mL) (1.73 × 10^3^ U·mg^−1^) and peak 3 (95–100 mL) (≈ 0.00 U·mg^−1^). The specific activity after each step of purification is shown in Table 3. BADE (40–50 mL, peak 2) was purified 9.55-fold with a 39.92% yield, and had a molecular mass of 22 kDa as determined by SDS-polyacrylamide gel electrophoresis (PAGE) (Figure 6).

### 2.7. Effect of pH on BADE Activity

**Figure 7:**
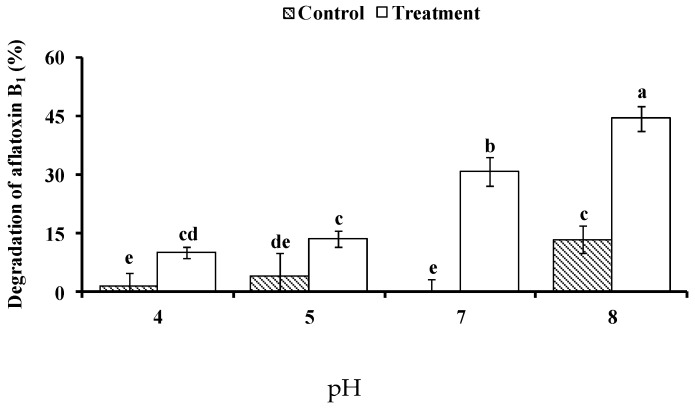
Effect of pH on AFB_1_ degradation by BADE from isolate L7. Values represent the means of three replicates and their standard errors. Control, AFB_1_ without BADE; treatment, AFB_1_ with BADE.

The extent of AFB_1_ degradation by BADE (40–50 mL, peak 2) from isolate L7 varied as a function of pH (Figure 7). There was no significant difference in AFB_1_ degradation among pH 4.0, 5.0, and 7.0 without BADE, and there was little degradation. Compared to the control (AFB_1_ without BADE), the degradation ratio of AFB_1_ in the presence of BADE increased significantly at pH 4.0 (10.40%), pH 5.0 (14.30%), and pH 7.0 (30.73%). The degradation ratio of AFB_1_ without BADE was 13.52% at pH 8.0 (shown in Figure 7), and the degradation ratio of AFB_1_ in the presence of BADE increased significantly from 13.52% to 44.70% at pH 8.0. The degradation ratio of AFB_1_ treated with BADE increased following the pH increase, and the degradation rate at pH 8.0 was the highest (31.18%) among different pH values, indicating that pH 8.0 is the optimal pH for BADE activity.

### 2.8. Effect of Metal Ions on BADE Activity

**Figure 8:**
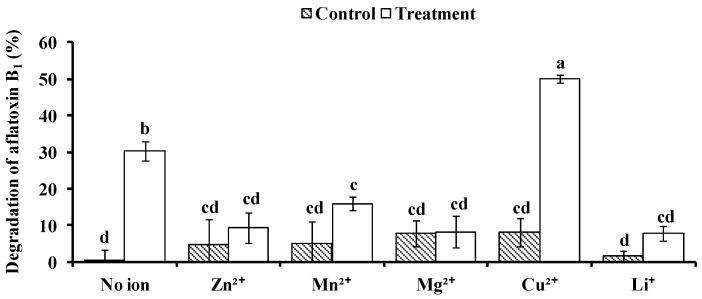
Effect of ions on AFB_1_ degradation by BADE from isolate L7. Values represent the means of three replicates and their standard errors. Control, AFB_1_ without BADE; treatment, AFB_1_ with BADE.

The effect of various metal ions on BADE (40–50 mL, peak 2) activity was evaluated. There was no significant difference in AFB_1_ degradation among the no-ion condition, Zn^2+^, Mn^2+^, Mg^2+^, Cu^2+^, and Li^+^ in the absence of BADE, and there was little degradation. Compared to the no-ion condition in the presence of BADE, BADE activity was inhibited by Zn^2+^, Mn^2+^, Mg^2+^, and Li^+^ (10 mmol·L^−1^) but was enhanced in the presence of Cu^2+^ (Figure 8).

### 2.9. Effect of Temperature on BADE Activity

**Figure 9:**
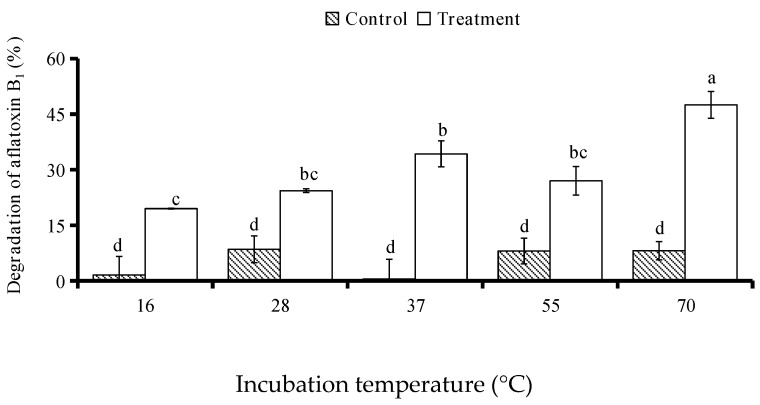
Effect of temperature on AFB_1_ degradation by BADE from isolate L7. The mixtures were incubated at indicated temperatures for 72 h. Values represent the means of three replicates and their standard errors. Control, AFB_1_ without BADE; treatment, AFB_1_ with BADE.

The AFB_1_-degrading activity of BADE (40–50 mL, peak 2) from isolate L7 varied as a function of temperature (Figure 9). There was no significant difference in AFB_1_ degradation among 16, 28, 37, 55, and 70 °C without BADE, and there was little degradation. Compared to the control (AFB_1_ without BADE), the degradation ratio of AFB_1_ in the presence of BADE increased significantly at 16 °C (19.50%), 28 °C (24.40%), 37 °C (34.27%), 55 °C (27.01%), and 70 °C (47.51%). The degradation ratio of AFB_1_ treated with BADE increased following the increase in temperature, and the degradation rate at 70 °C was the highest (47.51%) among different temperatures, indicating that 70 °C is the optimal temperature for BADE activity.

## 3. Discussion

This is the first study reporting that *B. shackletonii* has AFB_1_-degrading activity. *B. shackletonii* strain LMG 18435T, a strictly aerobic bacterium isolated from the eastern lava flow of northern Candlemas Island in the South Sandwich archipelago, was reported to be capable of decomposing large molecules into organic acids [25]. In addition, the thermophilic *B. shackletonii* strain K5 isolated from a biotrickling filter of a coal-fired power plant in Guangzhou, China showed a strong capacity for producing polyhydroxybutyrate [26].

We demonstrated that AFB_1_ in solution could be effectively degraded by the culture supernatant of *B. shackletonii* L7. This was similar to previous findings demonstrating that extracellular extracts from *R. erythropolis* liquid cultures degraded AFB_1_, with only 33.2% residual toxin after 72 h [17]. Incubation with 5 × 10^10^ resting cells/mL of *Fusarium aurantiacum* for 4 h completely removed AFM_1_ (8 µg·mL^−1^) [27]. Similar to *R. erythropolis* [17] and *S. maltophilia* 35-3 [19], degradation of AFB_1_ by the culture supernatant produced without pre-exposure to AFB_1_ showed the constitutive activity of *B. shackletonii* L7. The AFB_1_-degrading activity of the culture supernatant increased from 47.6% to 70.1% after ultrafiltration, and was decreased from 77.9% to 15.3% upon treatment with proteinase K and SDS, similar to the trends observed for the culture supernatants of *R. erythropolis*, *M. fulvus* ANSM068, and *S. maltophilia* [17,19,20]. These findings imply that the AFB_1_-degrading enzyme from *B. shackletonii* is an extracellular enzyme, in contrast to intracellular AFB_1_-degrading enzymes isolated from fungi [11,13].

The increase in AFB_1_ detoxification over time indicated that the AFB_1_-degrading enzyme from *B. shackletonii* was stable at 37 °C for one week. Similarly, a 66.8% reduction in AFB_1_ was observed over 72 h in the presence of *R. erythropolis* culture supernatant [28], whereas the AFB_1_ level was reduced by 70%–80% within 36 h and AFB_1_ was completely degraded after 72 h by liquid cultures of *Mycobacterium* strain FA4 [22]. AFB_1_ was quickly removed from liquid by binding to lactic acid bacteria, although some of the toxin was released over a prolonged period of incubation [10,19]. Binding may not be important in AFB_1_ reduction, given the continuous increase in detoxification by strain L7 over time. To further verify the role of binding (or adsorption) in the observed reduction in aflatoxin concentration, bacterial pellets of *B. shackletonii* L7 cultured for 24 h were washed twice with and resuspended in phosphate buffer (20 mM, pH 7.0). The resuspended liquid (0.026 g/mL) was sterilized at 121 °C for 20 min and was used for the AFB_1_ degradation test, using phosphate buffer rather than the resuspended liquid as a control. AFB_1_ concentration did not decrease relative to the control upon treatment with the resuspended liquid; the AFB_1_ degradation rate was 0% (data not shown). This indicates that binding does not play a role in AFB_1_ reduction.

Methods for purifying enzymes from microorganisms should be selected based on the activity and yield of the active component. In this study, crude AFB_1_-degrading enzymes were purified from the *B. shackletonii* culture supernatant by ion-exchange chromatography on a DEAE-sepharose column. AFB_1_-degrading enzymes have been purified from the fungi *A. tabescens* by ion-exchange chromatography and chromatofocusing chromatography [11], and from *P. ostreatus* by chromatography using DEAE-sepharose and phenyl sepharose columns [13]. In our study, BADE (40–50 mL, peak 2) with an estimated molecular mass of 22 kDa and the highest AFB_1_-degrading activity (3.85 × 10^3^ U·mg^−1^) was purified 9.55-fold from *B. shackletonii* cultures with a 39.92% yield, implying a low processing cost [29] which, along with the reasonably high overall yield [30], makes it suitable for large-scale production.

*B. shackletonii* BADE was active at a broad range of pH values (pH 4.0–8.0) and temperatures (16 °C–70 °C), which is advantageous for AF degradation in the digestive tracts of animals. Importantly, there was no decrease in enzymatic activity when the culture supernatant of strain L7 was subjected to heat treatment. This was in contrast with a previous study in which AFB_1_-degrading activity in the culture supernatant of *S. maltophilia* 35-3 was abolished by heat treatment [19]. These results imply that *B. shackletonii* BADE is heat-stable, similar to the enzyme in thermophilic *B. shackletonii* K5 [26].

The AFB_1_-degrading activity of *B. shackletonii* BADE was influenced by the presence of metal ions: the activity was enhanced by Cu^2+^ (10 mM) and inhibited by Zn^2+^, Mn^2+^, Mg^2+^, and Li^+^ (10 mM), in agreement with earlier reports [19,31]. We speculate that BADE is an oxidoreductase that utilizes Cu^2+^ as an activator. Cu^2+^ may participate in redox reactions in electron transport, transferring an oxygen atom to the AFB_1_ substrate; oxidized AFB_1_ would then be hydrolyzed into non-toxic products [32]. In contrast, 10 mM Zn^2+^ inhibited AFB_1_ degradation by BADE, which was similar to the one observed in *F. aurantiacum* [33]. Zn^2+^ may cause a conformational change in the enzyme that leads to its inactivation or decreases its affinity for AFB_1_ [28].

We evaluated the genotoxicity of AFB_1_ degradation products with the SOS Chromotest. The genotoxicity of AFB_1_ was significantly reduced by treatment with proteins or enzymes in *B. shackletonii* L7 culture supernatant in the presence of S9. It was previously demonstrated that treatment of AFB_1_ with enzymes altered the double bond of the furofuran ring, with consequent changes in the fluorescence and mutagenicity of the molecule [23]. In this study, BADE from strain L7 played a major role in AFB_1_ detoxification; we also showed that the chemical properties of AFB_1_ degradation products differed from those of the parent molecule, although the detailed mechanism of AFB_1_ degradation by *B. shackletonii* BADE has yet to be described.

To date, BADE from *B. shackletonii* is the only AF-degrading enzyme purified from a bacterium. Enzyme purification is the limiting factor in the industrial-scale production of AFs-degrading enzymes. Challenges associated with obtaining BADE by *B. shackletonii* fermentation include contamination of bacterial cultures and low yields. Nonetheless, our results suggest that treatment of food and feed with recombinant *B. shackletonii* BADE can potentially eliminate mycotoxin contamination.

## 4. Conclusions

We isolated *B. shackletonii* strain L7, which could degrade AFB_1_, AFB_2_, and AFM_1_. A presumed extracellular BADE was purified from strain L7 showed the highest activity at 70 °C and pH 8.0 in the presence of 10 mmol·L^−1^ Cu^2+^, and was the main protein involved in the detoxification of AFB_1_. These findings indicate that BADE from *B. shackletonii* L7 is a promising new agent for AF biodegradation. The cloning and expression of the BADE-encoding gene are currently underway in our laboratory.

## 5. Materials and Methods

### 5.1. Chemicals and Media

Standard solutions of AFB_1_, AFB_2_, AFG_1_, AFG_2_, and AFM_1_ were purchased from Sigma-Aldrich (St. Louis, MO, USA). Stock solutions (500 µg/L) were prepared by diluting the standard solutions with methanol. Coumarin medium (CM) was prepared as previously described [19]. NB was used for bacterial culture.

### 5.2. Collection of Bacteria

A total of 247 samples were collected from farm soils, maize, and rice in different provinces in China in June 2013. Bacterial strains with the ability to degrade AFB_1_ were isolated on CM medium [19]; selected colonies were further tested for AFB_1_-degrading activity.

### 5.3. Analysis of AFs Degradation

Isolates were cultured in NB for 12 h. For inoculation, a 12-h culture broth (1 mL) was transferred to NB (20 mL) and cultured at 37 °C with agitation for 24 h in a shaking incubator. AFB_1_ solution (500 µg/L; 0.1 mL) was added to 0.4 mL of microbial culture to obtain a final concentration of 100 µg/L. The detoxification test was performed in the dark at 37 °C without shaking for 72 h; sterile NB containing AFB_1_ at a final concentration of 100 µg/L was used as a control.

The reaction mixtures were extracted three times with chloroform according to a standard protocol [34]. The chloroform was evaporated under nitrogen gas at 60 °C and dried extracts were dissolved in 50% methanol in water (1:1, *v*/*v*), and the solution was passed through a sterile 0.22-µm pore size filter (Merck Millipore, Darmstadt, Germany) and analyzed by high-performance liquid chromatography using a C18 column (150 × 4.6 mm, 5 μm; Agilent Technologies, Santa Clara, CA, USA) and methanol:water (1:1, *v*/*v*) as the mobile phase at a flow rate of 1 mL/min. AFB_1_ was derived by a photochemical reactor (Waters, Milford, MA, USA) and detected with a fluorescence detector. Excitation and detection wavelengths were set at 360 and 440 nm, respectively. Percent AFB_1_ degradation was calculated using the formula (1 − AFB_1_ peak area in treatment/AFB_1_ peak area in control) × 100%. AFB_2_, AFG_1_, AFG_2_, and AFM_1_ degradations were analyzed in a similar manner.

### 5.4. Identification of Isolate L7

Biochemical and physiological analyses of isolate L7 were performed following standard protocols [35]. Genomic DNA of strain L7 was extracted using the TIANamp Bacterial DNA kit (Tiangen Biotech, Beijing, China) according to the manufacturer’s instructions. The 16S rRNA gene was amplified using a universal primer set consisting of 27F (5′-GAGAGTTTGATCCTGGCTCAG-3′) and 1492R (5′-TACCTTGTTACGACTT-3′) [36] and then sequenced. Similar sequences were identified by a BLAST search of the NCBI nucleotide database [37]. Strain L7 was preserved at −80 °C until use.

### 5.5. Degradation of AFB_1_ by Cell-Free Supernatant, Bacterial Cells, and Intracellular Cell Extracts of Strain L7

AFB_1_ degradation by cell-free supernatant, bacterial cells, and intracellular cell extracts of strain L7 was evaluated as previously described [16,19,38]. Briefly, 5 mL of fresh NB was inoculated with strain L7 at 37 °C with agitation at 180 rpm for 12 h. A 1-mL volume of the culture was transferred to 100 mL of the same medium. After cultivation at 37 °C with shaking at 180 rpm for 72 h, cells were harvested by centrifugation at 8000 rpm for 10 min at 4 °C. The culture supernatant was separated from the cell pellet and passed through a sterile 0.22-µm pore size filter and used for analysis of AFB_1_ degradation, as described above. NB was used rather than the supernatant as a control.

Bacterial cell pellets were washed twice with phosphate buffer (50 mM, pH 7.0) and resuspended in the same buffer. AFB_1_ degradation was evaluated as described above. Phosphate buffer was used rather than the bacterial cell suspensions as a control.

Cell pellets resuspended in phosphate buffer (50 mM, pH 7.0; 3 mL of buffer per gram of cell mass) were lysed on ice using an ultrasonic cell disintegrator (Ningbo Xinzhi Instruments, Ningbo, China). Cell lysates were centrifuged at 12,000× *g* for 10 min at 4 °C, and passed through a sterile 0.22-µm pore size filter. The AFB_1_ degradation test was performed as described above. Phosphate buffer was used rather than intracellular cell extracts as a control.

### 5.6. Effects of Incubation Period, Proteinase K + SDS, and Heat Treatment on AF Degradation by Strain L7 Culture Supernatant

The culture supernatant of strain L7 was prepared as described above. A 0.1-mL volume of AFB_1_ solution (500 µg/L) was combined with 0.4 mL of culture supernatant in a 10-mL tube, and the mixture was incubated in the dark at 37 °C without shaking. For incubation time studies, degradation was measured at 1, 6, 12, 24, 48, 72, 96, 120, and 168 h. The effect of heat treatment was assessed by immersing the culture supernatant in a boiling water bath for 10 min. The effect of proteinase K treatment with and without SDS was evaluated as previously described [19]. To determine the correlation between AFB_1_ degradation and protein concentration, an Amicon Ultra-15 centrifugal filter unit (Merck Millipore, Tullagreen, Iealand) with a cut-off molecular weight of 3 kDa was used for ultrafiltration of the culture supernatant to obtain different protein concentrations. NB alone processed in a similar manner and treated with AFB_1_ served as a control.

### 5.7. Genotoxicity Assay

The genotoxicity of AFB_1_ degradation products was evaluated using SOS Chromotest Basic S9 Activation Enzymes v.64 (Environmental Bio-detection Products, Mississauga, ON, Canada) according to the manufacturer’s instructions. Cell viability was monitored according to alkaline phosphatase activity at 420 nm using a microplate reader [39,40]. Sample concentrations are expressed as the percentage of sample volume in each well of a 96-well microtiter plate. AFB_1_ solution combined with NB was used as a positive control, AFB_1_ solution combined with culture supernatant of isolate L7 was used as a degraded sample, and methanol added to the culture supernatant of isolate L7 served as a negative control. After incubation in the dark at 37 °C for 72 h (at which point the AFB_1_ degradation ratio was 77.9% ± 2.3%), 10 µL of the diluted solution was dispensed into the appropriate wells, and a range of sample concentrations (100, 50, 25, 12.5, 6.25, and 3.125%) was tested using dimethyl sulphoxide as a diluent and reagent blank and 2-aminoanthracene serving as a positive control for S9.

### 5.8. Preparation of BADE

Large-scale *B. shackletonii* cultures were prepared in a 1-L working volume of NB (pH 7.4). A 10-mL volume of fresh NB was inoculated with strain L7 at 37 °C, with agitation at 180 rpm for 12 h; 10 mL of the culture was then transferred to 1 L of the same medium. After cultivation at 37 °C with shaking at 180 rpm for 72 h, cells were harvested by centrifugation at 8000 rpm for 10 min at 4 °C. The culture supernatant was separated from the cell pellet and passed through a sterile 0.22-µm pore size filter. The supernatant of the 1-L culture was heated for 10 min and then subjected to ultrafiltration with a cut-off molecular weight of 3 kDa at 5000× *g* for 20 min in a refrigerated centrifuge to obtain a final volume of 20 mL, yielding a sample that was concentrated 50-fold. The concentrated sample was dialyzed against 20 mmol·L^−1^ Tris-HCl buffer (pH 8.5) for 24 h at 4 °C.

### 5.9. Purification of B. shackletonii BADE

The dialyzed concentrated sample was subjected to ion-exchange chromatography on a DEAE-sepharose column (1.6 × 20 cm; GE Healthcare, Little Chalfont, UK). Proteins were packed in the column pre-equilibrated with 20 mmol·L^−1^ Tris-HCl buffer (pH 8.5) and were eluted with an increasing linear gradient (0%–100%) of 1 mol·L^−1^ NaCl with 20 mmol·L^−1^ Tris-HCl buffer (pH 8.5) at a flow rate of 2 mL·min^−1^ until no protein was detected. The flow-through and eluted enzyme fractions were collected and dialyzed for 24 h against 20 mmol·L^−1^ phosphate buffer (pH 7.4). Each fraction was concentrated by ultrafiltration with a cut-off molecular weight of 3 kDa; the final volumes of each fraction were 20 mL for Peak 1 (0–20 mL), 10 mL for Peak 2 (40–50 mL), 30 mL for Peak 2 (50–80 mL), 15 mL for Peak 3 (80–95 mL), and 5 mL for Peak 3 (95–100 mL). Protein concentration was determined by the Bradford method using Coomassie Brilliant Blue, and AFB_1_-degrading activity was evaluated as described above. After each fraction was incubated with AFB_1_ for 72 h, residual AFB_1_ was detected by high-performance liquid chromatography. To assess the degradation of AFB_1_ with the proteins in different peaks, AFB_1_ with 20 mmol·L^−1^ phosphate buffer (pH 7.4) was used as a control. One unit (U) of enzyme activity was defined as the amount of enzyme required to decrease the amount of AFB_1_ by 1 ng in 72h at 37 °C. This purification step yielded the active fraction, which was tested for AFB_1_-degrading activity. The molecular mass of the active fraction was verified by SDS-PAGE.

### 5.10. Determination of Molecular Mass

The molecular mass of the purified BADE from *B. shackletonii* L7 was determined by SDS-PAGE based on the method of Laemmli [41] on a 12% polyacrylamide gel with molecular weight markers (Thermo Fisher Scientific, Waltham, MA, USA). Protein bands were stained using the MS-Compatible Silver Stain kit (Tiandz, Beijing, China) according to the manufacturer’s protocol.

### 5.11. Characterization of BADE

#### 5.11.1. Determination of Optimum pH and Temperature for Enzymatic Activity

To determine the effects of pH on BADE activity, the initial pH value was adjusted to 4.0 and 5.0 with 0.1 mol·L^−1^ citrate phosphate, and to 7.0 and 8.0 with 0.1 mol·L^−1^ sodium phosphate. The effect of temperature on BADE activity was determined at 16, 28, 37, 55, and 70 °C in 0.1 mol·L^−1^ sodium phosphate buffer (pH 7.0). A 0.1-mL volume of AFB_1_ solution (500 µg/L) was added to 0.4 mL of BADE solutions with different pH and at different temperatures, with a solution at pH 7.0 and 37 °C used as a control along with solutions without BADE under the same conditions. The assays were performed in the dark at 37 °C without shaking for 72 h.

#### 5.11.2. Effect of Metal Ions on Enzyme Activity

To determine the effect of different metal ions on BADE activity, Zn^2+^ (ZnSO_4_), Mn^2+^ (MnCl_2_), Mg^2+^ (MgCl_2_), Cu^2+^ (CuSO_4_), and Li^+^ (LiCl) solutions at a final ion concentration of 10 mmol·L^−1^ were added to 0.1 mL of AFB_1_ (500 µg/L) combined with 0.4 mL of BADE solution (pH 7.0). BADE solution without any metal ions (no ion) was also used as a treatment. To assess the degradation of AFB_1_ in the presence of metal ions, solutions without BADE under the same conditions were used as controls. The assay was performed in the dark at 37 °C without shaking for 72 h.

### 5.12. Statistical Analysis

Differences between groups were evaluated by analysis of variance with the general linear model procedure in a completely randomized single-factor design using SAS software (SAS Institute, Cary, NC, USA). F tests were performed at the 0.05 level of probability. When a significant F value was detected, significant differences among the means were assessed with Duncan’s multiple range test.

## Figures and Tables

**Table 1:** Induction factors produced by samples in the SOS Chromotest.

Induction Factor ± Standard Deviation				
Concentration (%)	2-Aminoanthracene ^a^	Positive Control ^b^	Degraded Sample ^c^	Negative Control ^d^
100	3.49 ± 0.08	2.53 ± 0.06	1.06 ± 0.00	1.09 ± 0.05
50	3.02 ± 0.06	1.73 ± 0.14	1.02 ± 0.03	1.12 ± 0.10
25	2.29 ± 0.09	1.08 ± 0.06	1.00 ± 0.01	1.14 ± 0.09
12.5	1.78 ± 0.05	1.18 ± 0.09	1.02 ± 0.01	1.13 ± 0.04
6.25	1.42 ± 0.12	1.04 ± 0.05	0.96 ± 0.06	0.98 ± 0.09
3.125	1.45 ± 0.07	1.07 ± 0.08	1.07 ± 0.03	1.01 ± 0.11

^a^ Six two-fold dilutions of 2-aminoanthracene (initial concentration = 100 µg/mL) were prepared in 10% dimethyl sulphoxide/saline. A 10-µL volume of diluted 2-aminoanthracene was used as a positive S9 control. ^b^ A 0.06-mL volume of AFB_1_ solution (50 mg/L) was added to 1.44 mL of NB to obtain a final concentration of 2 mg/L. After incubation in the dark at 37 °C for 72 h, six two-fold dilutions were prepared, and a 10-µL volume was used as a positive control. ^c^ A 0.06-mL volume of AFB_1_ solution (50 mg/L) was added to 1.44 mL of culture supernatant of isolate L7 to obtain a final concentration of 2 mg/L. After incubation in the dark at 37 °C for 72 h (AFB_1_ degradation ratio = 77.9% ± 2.3%), six two-fold dilutions were prepared, and a 10-µL volume was used as the degraded sample. ^d^ A 0.06-mL volume of methanol was added to 1.44 mL of culture supernatant of isolate L7. After incubation in the dark at 37 °C for 72 h, six two-fold dilutions were prepared, and a 10-µL volume was used as a negative control.

**Table 2:** Degradation of AFB_1_ by L7 culture supernatant after 24 h of incubation.

Supernatant Conditions	Protein Concentration (mg/mL)	Degradation (%)
Culture supernatant	0.13 ± 0.03	47.58 ± 1.09
Culture supernatant with ultrafiltration ^a^	0.66 ± 0.04	70.12 ± 0.69
Boiled culture supernatant with ultrafiltration ^a^	0.10 ± 0.02	76.67 ± 0.85

^a^ The culture supernatant was concentrated by ultrafiltration with a cut-off molecular weight of 3 kDa.

**Table 3:** Summary of BADE purification.

Purification Step	Total Protein (mg) × 10^−3^	Total AFB_1_ Degradation Activity (U) *	Specific Activity (U·mg^−1^) × 10^2^ *	Purification (Fold)	Yield (%)
Culture filtrate	96.55	38.95	4.03	1.00	100.00
Peak 1 (0–20 mL)	1.35	3.55	26.30	6.53	9.10
Peak 2 (40–50 mL)	4.04	15.55	38.49	9.55	39.92
Peak 2 (50–80 mL)	6.37	15.21	23.88	5.93	39.05
Peak 3 (80–95 mL)	3.26	5.63	17.27	4.29	14.45
Peak 3 (95–100 mL)	0.20	5.5 × 10^−5^	0.0028	0.00069	0.00014

* One unit (U) of enzyme activity was defined as the amount of enzyme required to decrease the amount of AFB_1_ by 1 ng in 72 h at 37 °C.

## Supplementary Materials

Supplementary materials can be accessed at: www.mdpi.com/2072-6651/9/1/36/s1, Table S1: AFB_1_ degradation by individual microbial isolates selected using coumarin medium, Table S2: Characteristics of strain L7.
