# Supplementary material for: Novel Aflatoxin-Degrading Enzyme from Bacillus shackletonii L7
Source: Toxins (Basel). 2017 Jan 14;9(1):36. doi: 10.3390/toxins9010036 (PMC5308268; doi:10.3390/toxins9010036)
Supplement: Supplementary file 1 [file toxins-09-00036-s001.pdf]

Supplementary Material: Novel Aflatoxin-Degrading Enzyme from *Bacillus shackletonii* L7

Liang Xu, Mohamed Farah Eisa Ahmed, Lancine Sangare, Yueju Zhao, Jonathan Nimal Selvaraj, Fuguo Xing, Yan Wang, Hongping Yang and Yang Liu

Table S1. AFB_1 degradation by individual microbial isolates selected using coumarin medium.

No	Isolates [a]	Source of Samples	Percentage of Degradation (%) [b]
1	*Stenotrophomonas maltophilia* 97-D-5	Soil from Dongping, Taian	88.5
2	*Stenotrophomonas* sp. E-D-1	Soil from Dongping, Taian	86.4
3	*Stenotrophomonas maltophilia* 97-D-3	Soil from Dongping, Taian	85.0
4	*Pseudomonas aeruginosa* N17-1	Soil from Changping, Beijing	82.8
5	*Stenotrophomonas* sp. 97-D-1	Soil from Dongping, Taian	80.2
6	*Arthrobacter* sp. L15	Soil from Qiandou, Jilin	78.3
7	*Flavobacteriaceae* sp. 14	Soil from Quyang, Shanxi	74.1
8	*Pseudomonas aeruginosa* F26-1	Soil from Changping, Beijing	73.3
9	*Bacillus shackletonii* L7	Soil from Tangshan, Hebei	71.7
10	F26-2	Soil from Changping, Beijing	69.0
11	NS7	Maize from Yancheng, Jiangsu	65.4
12	NS3	Rice from Chaoyang, Beijing	65.4
13	19-A	Soil from Guangzhou, Guangdong	65.2
14	F18-1	Soil from Chaoyang, Beijing	56.1
15	NS10	Maize from Zhuozhou, Hebei	56.0
16	NS6	Maize from Changzhi, Shanxi	52.6
17	F24-C	Soil from Changping, Beijing	52.5
18	F24-1	Soil from Changping, Beijing	52.4
19	NS8	Maize from Baoji, Shannxi	45.5
20	NS9	Maize from Yancheng, Jiangsu	41.4
21	30	Soil from Shaoguan, Guangdong	39.3
22	F25	Soil from Changping, Beijing	38.4
23	NS1	Rice from Changping, Beijing	33.4
24	18	Soil from Fuyang, Anhui	30.3
25	NSL 25	Rice from Huaian, Jiangsu	30.0
26	16	Soil from Shantou, Guangdong	24.8
27	29-D-1	Soil from Quyang, Shanxi	22.7
28	20	Soil from Shaoguan, Guangdong	21.9
29	NSL24	Rice from Huaian, Jiangsu	21.8
30	M77	Maize from Suzhou, Anhui	21.4
31	M30	Maize from Suzhou, Anhui	19.5
32	NS2	Rice from Changping, Beijing	16.4
33	25-C	Soil from Shaoguan, Guangdong	16.0
34	25-B	Soil from Shaoguan, Guangdong	15.0
35	G-B	Soil from Shantou, Guangdong	15.8
36	NSL23	Rice from Yangzhou, Jiangsu	15.2
37	M15	Maize from Suzhou, Anhui	13.8
38	25-C-3	Soil from Fuyu, Jilin	13.1
39	25-C-2	Soil from Fuyu, Jilin	12.9
40	E-D	Soil from Guangzhou, Guangdong	12.7
41	11-B	Soil from Dianbai, Guangdong	11.6
42	F4-1	Soil from Changping, Beijing	11.0
43	M60	Maize from Yancheng, Jiangsu	10.8

[a] Individual microbial isolates grown on medium with coumarin as the sole carbon source; [b] The detoxification tests were conducted in the dark at 37 °C for 72 h. The percentage of AFB_1 degradation was calculated using the following formula: $(1 - AFB_1$ peak area in treatment/AFB_1 peak area in control$) \times 100\%$.

Table S2. Characteristics of strain L7.

**Item**	**Result** [a]	**Item**	**Result**	**Item**	**Result**	**Item**	**Result**
Morphological Characteristic:							
Cell shape	rod-shaped	Gram stain	+				
Physiological and Biochemical Characteristic:							
Oxidase activity	-	Arginine double hydrolase	-	Hydrolysis of gelatin	-	Hydrolysis of amylum	-
Catalase activity	+	Hydrolysis of butyrin	-	Nitrate reduction	-	Bile Esculin Test	+
Hydrolysis of casein	+	Hydrolysis of Tween 80	-	Production of Indole	-	Urease	-
Utilization of citrate	+	Production of H_2S	-	VP test	-	5% NaCl	+
Anaerobic growth	-	50 °C	+	55 °C	+	pH 9.0	+
Acid from:							
Glycerin	-	Erythritol	-	D-Arabinose	-	L-Arabinose	-
D-Ribose	+	D-Xylose	+	L-Xylose	-	Adonitol	-
β-Methy-D-Xyloside	-	D-Galactose	+	D-Glucose	+	D-Fructose	+
D-Mannose	+	L-Sorbose	-	L-Rhamnose	-	Dulcitol	-
Inositol	-	Mannitol	+	α-Methyl-D-Glucoside	-	*N*-Acetyl glucosamine	+
Sorbitol	-	α-Methyl-D-Mannopyranoside	-	Amygdalin	+	Arbutin	+
Aesculin	+	Salicin	+	D-(+)-Cellobiose	+	D-Maltose	+
D-Lactose	+	D-(+)-Melibiose	-	D-Sucrose	+	D-Trehalose	+
Inulin	-	D-Melezitose	-	D-Raffinose	-	Amylum	-
Glycogen	-	Xylitol	-	D-Lyxose	-	D-Fucose	-
L-Fucose	-	D-Arabitol	-	L-Arabitol	-	Potassium gluconate	-
2-Keto-D-Gluconate	-	5-Keto-D-Gluconate	-				

[a] "+" means positive response; "−" means negative response.
